# A prospective cohort study on risk factors of musculoskeletal complaints (pain and/or stiffness) in a general population. The Tromsø study

**DOI:** 10.1371/journal.pone.0181417

**Published:** 2017-07-20

**Authors:** Ole Fredrik Andorsen, Luai Awad Ahmed, Nina Emaus, Elise Klouman

**Affiliations:** 1 Department of Community Medicine, Faculty of Health Sciences, University of Tromsø – The Arctic University of Norway, Tromsø, Norway; 2 Department of Health and Care Sciences, Faculty of Health Sciences, University of Tromsø – The Arctic University of Norway, Tromsø, Norway; 3 Institute of Public Health, College of Medicine and Health Sciences, United Arab Emirates University, Al Ain, United Arab Emirates; Universite de Nantes, FRANCE

## Abstract

**Background:**

Female gender has been associated with musculoskeletal complaints (MSCs), but there are limited studies on how other factors may influence women and men differently. The aim of this prospective cohort study was to explore possible predictors of MSCs in women and men free of MSCs at baseline.

**Methods:**

The present study included participants from the population-based Tromsø study, with baseline data from 1994–1995 and follow-up data from 2007–2008. MSCs were defined as having pain and/or stiffness in muscles and joints for 3 consecutive months during the past year. Predictors of MSCs were examined through binary logistic regression analyses and presented as odds ratios with 95% confidence intervals.

**Results:**

At baseline 4,496 participants reported no MSCs and among these 2,015 (44.8%) and 441 (9.8%) participants reported mild or severe MSCs, respectively, at follow-up. Female gender predicted MSCs in multivariable logistic regression analyses (odds ratio [OR] 1.46, 95% confidence interval [CI]: 1.29–1.66). Educational level of primary/secondary school (OR 1.73, 95% CI: 1.46–2.05) was the strongest predictor of MSCs, followed by poor self-perceived health (OR 1.62, 95% CI: 1.30–2.02). Other predictors were BMI ≥30 kg/m^2^ (OR 1.39, 95% CI: 1.10–1.77) and smoking (OR 1.33, 95% CI: 1.16–1.52). Age and physical activity level were not significantly associated with MSCs. Gender-stratified analyses revealed that mental health complaints (i.e., depression and/or anxiety) predicted MSCs in men (OR 2.03, 95% CI: 1.18–3.50), but not in women. Current smoking (OR 1.43, 95% CI: 1.16–1.76) and poor self-perceived health (OR 1.90, 95% CI: 1.34–2.71) showed slightly higher odds ratios among women than men, but the gender differences were not significant.

**Conclusion:**

The present study demonstrates that several negative health determinants are predicting subsequent MSCs. However, the examined risk factors could not explain the higher prevalence of MSCs in women.

## Background

Chronic musculoskeletal complaints (MSCs) are recognised as a major and costly health problem in Norway [1] and other Western countries [2]. Patients with symptoms such as pain and/or stiffness from the musculoskeletal system constitute a large portion of general practice patients and contribute heavily to absence from work due to sickness and disability pension in Norway [3–5]. The prevalence of MSCs is increasing in many populations and in younger age groups [6–8]. Several characteristics such as age, gender, socioeconomic status, smoking, overweight, physical inactivity, general health, and mental health have previously been associated with MSCs [2, 6, 9–17], but findings have been inconsistent and many of the studies were cross-sectional. Some prospective studies have evaluated these associations and found that several of the above-mentioned characteristics can predict the risk of MSCs in general populations [9, 10, 18, 19].

Many studies have reported gender differences in the prevalence of MSCs [6, 14, 15, 20–22], while others have not [13, 23, 24]. For example, our previous cross-sectional study of MSCs in Northern Norway showed a higher prevalence of MSCs among women than in men [25]. The same study also indicated that factors associated with MSCs (self-perceived health, smoking, obesity, and physical activity) could display different effects in women and men. The same phenomenon has been observed for other conditions: in a large prospective study from Norway, smoking was a stronger predictor of colorectal cancer in women than men [26]. If women and men respond differently to risk factors, it may explain the gender difference in the prevalence of MSCs.

Using data from 13 years of follow-up, the aim of the present study was to determine which factors predicted MSCs in a prospective cohort of Northern Norwegian adult women and men with no MSCs at baseline.

## Methods

### The Tromsø study

The Tromsø study is a longitudinal, population-based, multi-purpose health study carried out in the municipality of Tromsø, Northern Norway. The study currently consists of seven health surveys (Tromsø 1–7); Tromsø 1 was performed in 1974 and Tromsø 7 was completed at the end of 2016. The participation rate for all six surveys has ranged from 65% to 77% [27]. All participants of the Tromsø study are asked to complete questionnaires and attend medical examinations.

The present analyses used data from Tromsø 4 (1994–1995) and Tromsø 6, as information on MSCs in these surveys was comparable. Tromsø 4 included men and women aged 25–97 years. All inhabitants of Tromsø, Northern Norway, older than 25 years (37,558) were invited and 27,158 attended (12,865 men and 14,293 women). After excluding those who had moved away or died, the participation rate for Tromsø 4 was 77%. Tromsø 6 included men and women aged 30–87 years; of the 19,762 invited, 12,984 (66%) attended [27]. In total, 10,326 individuals attended both Tromsø 4 and Tromsø 6.

### Variables and classifications

Chronic MSCs were defined as pain and/or stiffness in muscles and joints lasting for at least 3 consecutive months during the previous year (hereafter referred to as simply MSCs). Information on MSCs in Tromsø 4 (baseline) was collected through the screening question, “Have you during the last year suffered from pain and/or stiffness in muscles and joints that have lasted continuously for at least 3 months?” (yes/no). Respondents who answered “no” were categorised as not having MSCs. The reliability of this screening question has been evaluated to be good [6]. The Tromsø 6 questionnaire (follow-up) did not include only one MSCs variable. Instead, information on MSCs at follow-up was composed from a questionnaire that consisted of six variables, one for each of the body regions: neck/shoulder, arm/hand, upper back, lumbar back, hip/leg/feet, and other muscles and joints. The participants were asked if during the last year they suffered from pain and/or stiffness in muscles and joints in different body regions that lasted for at least three consecutive months, i.e., identical to the phrasing at baseline. These six questions had three possible replies: “no complaints”, “mild complaints” and “severe complaints” and for descriptive purposes information from the six variables were merged to compute one MSCs variable with the same three outcomes [25]. For logistic regression analyses we merged information from the six variables to compute the following dependent variables: 1) Overall MSCs were defined as having mild and/or severe complaints in one or more body region versus no complaints. 2) Severe MSCs were defined as having only severe complaints in one or more body region versus no and/or mild complaints. 3) Based on previous prevalence calculations [25], we computed a variable on multi-region MSCs [22, 23, 28, 29], defined as having ≥3 affected body regions versus fewer or no body regions affected. 4) Body region-specific analyses were performed on each of the six MSCs variables separately with mild and severe complaints merged versus no complaints.

#### Independent variables

The baseline risk factors included age, gender, marital status, current smoking, self-perceived health, mental health complaints, education level, body mass index (BMI), and leisure time physical activity. Information on these factors was collected through questionnaires, except for BMI, which was calculated from data on height and weight measured in light clothing and without shoes. Three categories of BMI were created (BMI ≤24.9 kg/m^2^, 25–29.9 kg/m^2^ and ≥30 kg/m^2^). Participants were categorised as married if they had answered either “married” or “registered partnership”. The question on self-perceived health had four alternatives: very good, good, not so good and poor, merged into two categories: “poor” or “good”. Those reporting current cigarette, cigar, or pipe smoking at baseline were categorised as current smokers. Information on mental health was taken from the CONOR-Mental Health Index (MHI), a 7-item questionnaire. Questions were answered using a 4-level scale, ranging from “no” (1) to “very” (4). Thus, the total score for all seven questions ranged from 7–28. The CONOR-MHI average score was calculated by dividing the total score by the number of items, i.e., seven. Based on a validation study, the cut-off was set to 2.15, creating two groups: CONOR-MHI <2.15 and CONOR-MHI ≥2.15, i.e. indicating mental health complaints [30]. Educational level was categorised as primary/secondary school, technical school, high school, and college/university. Physical activity level was determined from two questions, one on light activity (not sweating/out of breath) and one on hard physical activity (sweating/out of breath). The responses to these questions were combined into a four-category physical activity index: sedentary (0 hours/week), low (<3 hours/week), moderate (3–5 hours/week) and high (≥6 hours/week) [31]. This index is in concordance with the Norwegian Directorate of Health’s recommendations of 30 minutes of daily physical activity for adults [32]. The questionnaires are available on the Tromsø Study’s website (https://en.uit.no/prosjekter/prosjekt?p_document_id=80172) [33].

### Study sample

**Fig 1:**
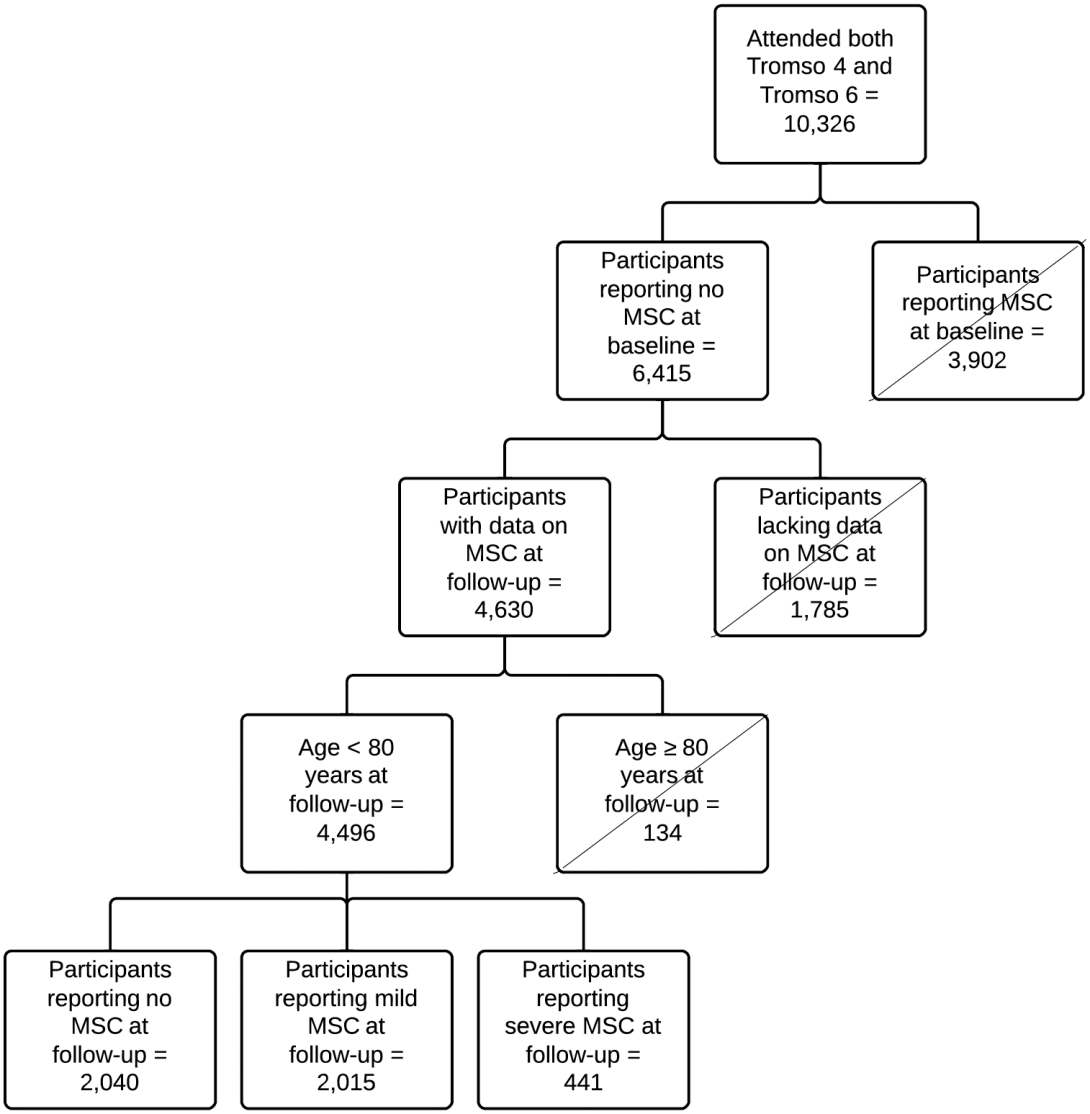
Flow chart presenting participants attending both surveys^1^ and those selected for the present study^2^. ^1^The Tromsø Study surveys from 1994–95 (Tromsø 4; baseline) and 2007–08 (Tromsø 6; follow-up), ^2^A prospective cohort study of an arctic general population consisting of 4,496 adult men and women free of MSCs at baseline, MSCs: musculoskeletal complaints (pain and/or stiffness in muscles and joints for 3 consecutive months during the past years).

In total, 10,326 individuals attended both baseline and follow-up. In order to create valid information on MSCs, 3,902 individuals who reported MSCs or had missing information on MSCs (n = 9) at baseline were excluded, as were those with missing answers on one or more of the six MSCs variables at follow-up (n = 1,785). Because of low participation rates in the age groups 80–84 years (42.4%) and 85–87 years (30.5%) at follow-up, we excluded these age groups (n = 134). Thus, 4,496 participants reporting absence of MSCs at baseline were included in this study (Fig 1).

### Statistics

Differences in the baseline characteristics of participants with no complaints, mild complaints, and severe complaints were analysed using ANOVA for continuous variables, and the Pearson chi-square test for categorical variables. Bonferroni corrections were conducted to adjust for multiple comparisons. The influence of independent variables on MSCs was evaluated using binary logistic regression to estimate odds ratios (OR) with 95% confidence intervals (CI) and p-values for trend analyses. Logistic regression analyses were first performed using the dichotomised dependent variable on overall MSCs (mild/severe MSCs versus no MSCs). Logistic regression was then performed on the dichotomised dependent variable on only severe MSCs. Predictors of multiregional MSCs were identified in a regression model using MSCs in ≥3 regions versus <3 regions as the dependent variable. Finally, we evaluated predictors of MSCs for each body region separately. Crude logistic regression analyses were first performed and then adjusted for age and gender. Covariates that showed a significant relationship in the age- and gender-adjusted models were included in multivariable models. Finally, logistic regression analyses including interaction terms were performed to examine gender differences in predictors of MSCs and to assess the significance level.

Missing values on independent variables at baseline were: marital status (n = 6), smoking status (n = 7), BMI (n = 5), self-perceived health (n = 2), mental health (n = 67), educational level (n = 8), and physical activity level (n = 40). Thus of the 4,496 participants in this study, 4,373 were included in the multivariable regression models. The statistical analyses were performed by SPSS version 21. All tests were performed two-sided and a p-value <0.05 was considered significant.

### Ethics

The Regional Committee for Medical and Health Research Ethics and the Norwegian Data Inspectorate approved the Tromsø study, and written informed consent was obtained from all participants.

## Results

### Descriptive statistics

The study sample consisted of 46.6% women. Mean age at baseline in 1994–1995 was 43.2 years. 31.7% of participants reported current smoking at baseline, and nearly one out of ten reported poor self-perceived health. Mean BMI was 24.8 kg/m^2^ and 8.0% were obese (BMI ≥30 kg/m^2^). Nearly one out of four (23.4%) reported primary/secondary school as their highest level of education (Table 1). At follow-up in 2007–2008, 44.8% of the study sample reported mild MSCs (women 46.8% and men 43.1%) and 9.8% reported severe MSCs (women 12.0% and men 7.9%).

**Table 1:** Baseline characteristics by category of MSCs^1^ after 13 years of follow-up. The Tromsø study^2^.

Risk factors at baseline	Total population	Men (n = 2.400)				Women (n = 2,096)			
		MSCs^1^				MSCs^1^			
	(n = 4,496)	None (n = 1,176)	Mild (n = 1,035)	Severe (n = 189)	*p*^3^	None (n = 864)	Mild (n = 980)	Severe (n = 252)	*p*^3^
Age, years (mean ± SD)	43.2 (11.0)	44.0 (10.7)	45.1 (10.7)	43.3 (9.9)	0.018	41.5 (11.2)	42.7 (11.1)	39.8 (10.8)	0.001
Not married, N (%)	1,712 (38.1)	440 (37.4)	361 (34.9)	73 (38.6)	0.389	353 (40.9)	362 (36.9)	123 (48.8)	0.002
Current smoking, N (%)	1,425 (31.7)	338 (28.8)	346 (33.5)	78 (41.3)	0.001	226 (26.2)	331 (33.8)	106 (42.2)	<0.001
Poor self-perceived general health, N (%)	446 (9.9)	90 (7.7)	117 (11.3)	37 (19.6)	<0.001	53 (6.1)	104 (10.6)	45 (17.9)	<0.001
Mental health complaints^4^ N (%)	157 (3.5)	20 (1.7)	37 (3.6)	8 (4.3)	0.010	33 (3.9)	39 (4.0)	20 (8.2)	0.011
**Educational level**									
Primary/secondary, N (%)	1052 (23.4)	221 (18.8)	273 (26.4)	50 (26.6)	<0.001	164 (19.0)	275 (28.1)	69 (27.4)	<0.001
Technical School, N (%)	1254 (27.9)	305 (26.0)	310 (30.0)	75 (39.9)	<0.001	211 (24.4)	275 (28.1)	78 (31.0)	0.064
High School, N (%)	451 (10.0)	114 (9.7)	69 (6.7)	14 (7.4)	0.033	110 (12.7)	109 (11.2)	35 (13.9)	0.386
College/university, N (%)	1731 (38.6)	535 (45.5)	381 (36.9)	49 (26.1)	<0.001	378 (43.8)	318 (32.5)	70 (27.8)	<0.001
**Body mass index (kg/m**^**2**^**)**									
≤24,9, N (%)	2456 (54.7)	553 (47.1)	436 (42.1)	69 (36.5)	0.006	630 (73.0)	607 (62.0)	161 (64.1)	<0.001
25,0–29,9, N (%)	1675 (37.3)	530 (45.1)	503 (48.6)	95 (50.3)	0.175	181 (21.0)	296 (30.2)	70 (27.9)	<0.001
≥30, N (%)	360 (8.0)	91 (7.8)	96 (9.3)	25 (13.2)	0.039	52 (6.0)	76 (7.8)	20 (8.0)	0.293
**Physical activity level**									
Sedentary, N (%)	241 (5.4)	63 (5.4)	51 (5.0)	20 (10.6)	0.008	34 (4.0)	50 (5.1)	23 (9.2)	0.004
Low, N (%)	1,837 (41.2)	468 (40.1)	434 (42.5)	77 (41.0)	0.523	342 (39.9)	408 (42.0)	108 (43.4)	0.519
Moderate, N (%)	2,054 (46.1)	518 (44.3)	443 (43.3)	75 (39.9)	0.511	430 (50.2)	476 (49.0)	112 (45.0)	0.353
High, N (%)	324 (7.3)	119 (10.2)	94 (9.2)	16 (8.5)	0.635	51 (6.0)	38 (3.9)	6 (2.4)	0.025

^1^Musculoskeletal complaints (pain and/or stiffness in muscles and joints lasting at least 3 months during the past year),

^2^A prospective study of an arctic general population consisting of 4,496 adult men and women free of MSCs at baseline,

^3^Pearson chi-square for categorical variables and ANOVA for continuous variables (Bonferroni adjusted significance level: p-value < 0.003).

^4^Cohort of Norway Mental Health Index ≥2.15.

SD: standard deviation, BMI: body mass index.

### Overall musculoskeletal complaints

Table 2 shows the findings from the age- and gender-adjusted and the gender-stratified age-adjusted logistic regression analyses. With the exception of marital status, all other covariates showed significant associations with MSCs in the total population. In addition, smoking, poor self-perceived health, low education, and high BMI were significantly associated with MSCs in the gender-stratified models. Lower physical activity levels were associated with MSCs only in women, and mental health complaints increased MSCs risk only in men.

**Table 2:** Predictors of MSCs^1^ in age- and gender-adjusted regression models in a general population of Norway^2^.

	Odds ratio^3^ (95% Confidence interval)		
	Total	Men	Women
Age (5 years age groups)	**1.00 (1.00–1.01)**	1.01 (1.00–1.02)	1.01 (1.00–1.01)
Gender (Women versus men)	**1.39 (1.24–1.57)**		
Marital status (not married versus married)	0.97 (0.85–1.11)	0.98 (0.82–1.17)	0.97 (0.80–1.16)
Current smoking (yes versus no)	**1.43 (1.26–1.63)**	**1.32 (1.11–1.57)**	**1.58 (1.30–1.91)**
Self-perceived general health (poor versus good)	**1.85 (1.49–2.28)**	**1.69 (1.28–2.23)**	**2.08 (1.50–2.90)**
Mental health complaints^4^	**1.61 (1.15–2.25)**	**2.24 (1.31–3.82)**	1.27 (0.82–1.96)
**Educational level**			
Primary/secondary	1.91 (1.63–2.25)*	**1.80 (1.45–2.23)**	**2.11 (1.65–2.69)**
Technical school	1.60 (1.38–1.85)*	**1.57 (1.29–1.91)**	**1.65 (1.32–2.06)**
High School	1.09 (0.88–1.35)	0.92 (0.67–1.26)	1.25 (0.94–1.67)
College/university	1.00	1.00	1.00
**Body mass index (kg/m**^**2**^**)**			
≤24.9	1.00	1.00	1.00
25.0–29.9	1.37 (1.20–1.56)*	**1.21 (1.02–1.44)**	**1.65 (1.34–2.03)**
≥30	**1.48 (1.18–1.86)**	**1.44 (1.07–1.94)**	**1.50 (1.05–2.15)**
*p-value for trend*	**p < 0.001**	**p = 0.004**	**p < 0.001**
**Physical activity level**			
Sedentary	**1.55 (1.10–2.17)**	1.21 (0.79–1.85)	**2.45 (1.38–4.35)**
Low	**1.32 (1.04–1.67)**	1.17 (0.88–1.56)	**1.73 (1.13–2.65)**
Moderate	1.20 (0.95–1.52)	1.08 (0.81–1.43)	**1.57 (1.03–2.40)**
High	1.00	1.00	1.00
*p-value for trend*	**p = 0.002**	p = 0.17	**p = 0.003**

^1^Musculoskeletal complaints (Mild/severe pain and/or stiffness in muscles and joints lasting at least 3 months during the past year),

^2^The Tromsø Study; a prospective study of an arctic general population consisting of 4,496 adult men and women free of MSCs at baseline,

^3^Binary regression, adjusted for age and gender (bold text = significant result),

^4^Cohort of Norway Mental Health Index ≥2.15. OR: odds ratio,

*significant interaction with gender; p<0.05.

The associations were similar in the multivariable regression analysis of mild/severe versus no MSCs (Table 3). Female gender predicted MSCs (OR 1.46, 95% CI: 1.29–1.66), as did current smoking (OR: 1.33, 95% CI: 1.16–1.52), poor self-perceived health (OR: 1.62, 95% CI: 1.30–2.02), an educational level of primary/secondary (OR: 1.73, 95% CI: 1.46–2.05), and a BMI ≥30 kg/m^2^ (OR: 1.39, 95% CI: 1.10–1.77), with odds ratios that were slightly higher in women. Mental health complaints were not significantly predicting MSCs in this multivariable analysis of the total population. However, after stratification for gender, mental health complaints were shown to predict MSCs in men (OR 2.03, 95% CI: 1.18–3.50), but not in women (OR 0.95, 95% CI: 0.60–1.51). The interaction term analysis with gender and mental health complaints did almost reach the significance level of 5% (p = 0.051). Furthermore, BMI ≥30 kg/m^2^ increased the risk of MSCs in men (OR 1.39, 95% CI: 1.02–1.89), but not in women (OR 1.35, 95% CI: 0.93–1.95). However, the interaction term analysis with gender and BMI ≥30 kg/m^2^ was not significant (p = 0.537), as were the interaction terms involving the other covariates analysed in this multivariable regression model. Finally, reporting a sedentary physical activity level did not predict MSCs in this multivariable analysis.

**Table 3:** Predictors of MSCs^1^ in multivariable regression models in a general population of Norway^2^.

	Odds ratio^3^ (95% Confidence interval)		
	Total	Men	Women
Age (5 years age groups)	1.00 (0.99–1.00)	1.00 (0.99–1.01)	0.99 (0.98–1.00)
Gender (Women versus men)	**1.46 (1.29–1.66)**		
Current smoking (yes versus no)	**1.33 (1.16–1.52)**	**1.24 (1.03–1.48)**	**1.43 (1.16–1.76)**
Self-perceived general health (poor versus good)	**1.62 (1.30–2.02)**	**1.46 (1.09–1.94)**	**1.90 (1.34–2.71)**
Mental health complaints ≥2.15^4^	1.34 (0.94–1.90)	**2.03 (1.18–3.50)**	0.95 (0.60–1.51)
**Educational level**			
Primary/secondary	**1.73 (1.46–2.05)**	**1.66 (1.32–2.07)**	**1.83 (1.41–2.38)**
Technical school	**1.51 (1.30–1.76)**	**1.52 (1.24–1.86)**	**1.54 (1.21–1.94)**
High school	1.02 (0.82–1.27)	0.88 (0.64–1.21)	1.16 (0.86–1.55)
College/university	1.00	1.00	1.00
**Body mass index (kg/m**^**2**^**)**			
≤24.9	1.00	1.00	1.00
25.0–29.9	1.36 (1.19–1.56)*	**1.22 (1.02–1.45)**	**1.62 (1.30–2.01)**
≥30	**1.39 (1.10–1.77)**	**1.39 (1.02–1.89)**	1.35 (0.93–1.95)
*p-value for trend*	**p < 0.001**	**p = 0.017**	**p < 0.001**
**Physical activity**			
Sedentary	1.16 (0.82–1.66)	1.00 (0.64–1.55)	1.61 (0.88–2.95)
Low	1.19 (0.93–1.52)	1.10 (0.82–1.48)	1.48 (0.91–2.18)
Moderate	1.19 (0.93–1.51)	1.08 (0.81–1.45)	1.45 (0.94–2.23)
High	1.00	1.00	1.00
*p-value for trend*	p = 0.501	p = 0.804	p = 0.417

^1^Musculoskeletal complaints (Mild/severe pain and/or stiffness in muscles and joints lasting at least 3 months during the past year),

^2^The Tromsø Study; a prospective study of an arctic general population consisting of 4,496 adult men and women free of MSCs at baseline,

^3^Multivariable logistic regression analyses, the models included all variables listed *(bold text = significant result)*,

^4^Cohort of Norway Mental Health Index ≥2.15. OR: odds ratio,

*significant interaction with gender; p<0.05

**Fig 2:**
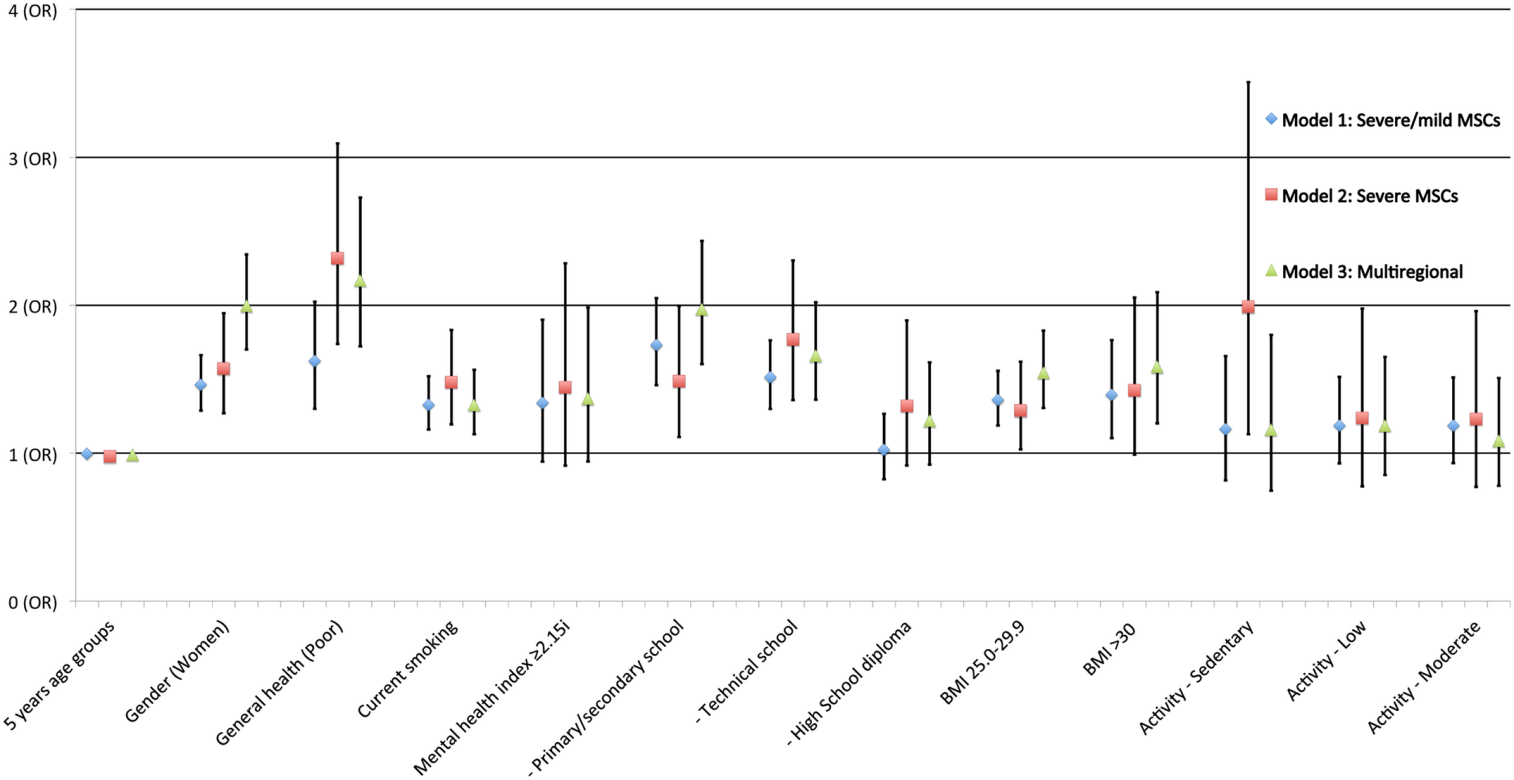
Graphical presentation of the relationship^1^ between health determinants and MSCs^2^. The Tromsø study^3^. ^1^Graphical presentation of multivariable logistic regression models, ^2^Musculoskeletal complaints (pain and/or stiffness in muscles and joints for 3 consecutive months during the past years); Model 1: MSCs = severe/mild versus no MSCs, model 2: MSCs = severe versus no and mild MSCs, model 3: MSCs in ≥3 body regions versus < 3 body regions, ^3^ A prospective cohort study of an arctic general population through 13 years consisting of 4,496 adult men and women free of MSCs at baseline.

The results of the multivariable analysis on severe (model 2) and multiregional MSCs (model 3) compared to severe and mild together (model 1) are shown in Fig 2. The figure shows that female gender, current smoking, poor self-perceived health, low educational level, and high BMI are consistent predictors of MSCs in the three different models.

### Body region-specific musculoskeletal complaints

Female gender, poor self-perceived health, and an educational level of primary/secondary or technical school increased the risk of reporting MSCs in all six body regions (Table 4). Mental health complaints increased the risk of MSCs in the lumbar back (OR 1.70, 95% CI: 1.21–2.38), hip/leg/feet (OR 1.51, 95% CI: 1.07–2.15), and other muscles and joints (OR 1.81, 95% CI: 1.01–3.23). Having a BMI ≥30 kg/m^2^ at baseline strongly predicted MSCs of the hip/leg/feet (OR 1.74, 95% CI: 1.36–2.24). Physical activity level was not significantly associated with MSCs in the upper back, lumbar back, hip/leg/feet, and other regions.

**Table 4:** Predictors of MSCs^1^ after 13 years of follow-up in an adult general population of Norway^2^.

	Odds ratio^3^ (95% confidence interval)					
	Neck/Shoulders	Arms/Hands	Upper back	Lumbar back	Hip/Leg/Feet	Other regions
Age (5-year age groups)	**0.98 (0.98–0.99)**	1.00 (0.99–1.00)	**0.97 (0.96–0.98)**	1.00 (0.99–1.00)	**1.01 (1.01–1.02)**	1.00 (0.99–1.02)
Gender (women versus men)	**1.58 (1.38–1.81)**	**1.84 (1.58–2.15)**	**2.21 (1.83–2.67)**	**1.16 (1.00–1.33)**	**1.95 (1.68–2.25)**	**1.67 (1.24–2.25)**
Current smoking (yes versus no)	**1.18 (1.03–1.36)**	**1.30 (1.11–1.52)**	**1.28 (1.06–1.54)**	**1.35 (1.17–1.57)**	**1.36 (1.17–1.58)**	1.29 (0.96–1.75)
Self-perceived general health (poor versus good)	**1.81 (1.46–2.24)**	**1.87 (1.49–2.35)**	**2.44 (1.89–3.16)**	**1.47 (1.18–1.84)**	**1.82 (1.46–2.27)**	**1.79 (1.21–2.66)**
Mental health complaints^4^	1.14 (0.81–1.61)	0.91 (0.61–1.35)	1.16 (0.76–1.77)	**1.70 (1.21–2.38)**	**1.51 (1.07–2.15)**	**1.81 (1.01–3.23)**
**Educational level**						
Primary/secondary school	**1.69 (1.42–2.02)**	**1.75 (1.43–2.15)**	**1.84 (1.44–2.35)**	**1.45 (1.20–1.74)**	**1.60 (1.33–1.94)**	**2.15 (1.45–3.18)**
Technical school	**1.60 (1.36–1.88)**	**1.60 (1.32–1.94)**	**1.46 (1.16–1.84)**	**1.43 (1.21–1.70)**	**1.39 (1.17–1.66)**	**1.64 (1.11–2.41)**
High School	1.12 (0.89–1.41)	1.20 (0.92–1.57)	1.15 (0.84–1.58)	1.03 (0.80–1.33)	1.02 (0.79–1.33)	1.43 (0.83–2.44)
University	1.00	1.00	1.00	1.00	1.00	1.00
**Body mass index (kg/m**^**2**^**)**						
≤24.9	1.00	1.00	1.00	1.00	1.00	1.00
25–29.9	**1.26 (1.10–1.46)**	**1.33 (1.13–1.57)**	**1.50 (1.24–1.83)**	**1.35 (1.16–1.57)**	**1.47 (1.26–1.71)**	**1.59 (1.17–2.17)**
≥30	**1.34 (1.05–1.70)**	**1.48 (1.13–1.93)**	**1.62 (1.18–2.22)**	1.05 (0.81–1.36)	**1.74 (1.36–2.24)**	1.36 (0.81–2.28)
**Physical activity**						
Sedentary	1.36 (0.93–1.98)	**1.76 (1.16–2.69)**	1.23 (0.74–2.05)	1.08 (0.73–1.58)	0.86 (0.57–1.30)	0.75 (0.33–1.74)
Low	**1.38 (1.05–1.81)**	1.22 (0.88–1.70)	1.17 (0.80–1.73)	1.00 (0.76–1.33)	1.15 (0.86–1.54)	0.91 (0.49–1.66)
Moderate	1.31 (0.99–1.71)	1.28 (0.92–1.77)	1.04 (0.71–1.54)	1.07 (0.82–1.41)	0.99 (0.74–1.33)	1.04 (0.57–1.89)
High	1.00	1.00	1.00	1.00	1.00	1.00

^1^Musculoskeletal complaints (Mild/severe pain and/or stiffness in muscles and joints in different body regions lasting at least 3 months during the past year),

^2^The Tromsø Study; a prospective cohort study of an arctic general population consisting of 4,496 adult men and women free of MSCs at baseline,

^3^Multivariable logistic regression analyses, the models included all variables listed (bold text = significant result),

^4^Cohort of Norway Mental Health Index ≥2.15.

### Effect of excluding participants with missing information on musculoskeletal complaints

To evaluate the consequences of excluding participants with missing values on one or more of the MSCs variables at follow-up, we compared the baseline characteristics of the study sample (n = 4,496) and the excluded population (n = 1,785). Excluded participants were more likely to be female (54.7% versus 46.6%, p<0.001), have a BMI ≥30 kg/m^2^ (11.0% versus 8.0%, p<0.001), primary/secondary school as their highest level of education (41.7% versus 23.4%, p<0.001), poorer self-perceived health (17.7% versus 9.9%, p<0.001), and to be current smokers (33.8% versus 31.7%, p = 0.012). Furthermore, the excluded population was more likely to report a sedentary physical activity level than the study sample (8.0% versus 5.3%, p<0.001). Marital status and high physical activity level were similar in the two populations. A sensitivity analysis was performed using logistic regression models in which all 6,415 participants reporting no MSCs at baseline were included. Those who had reported MSCs in any region at follow-up were included among participants with overall MSCs and those who did not report MSCs at follow-up were coded as no MSCs (S1 and S2 Appendices). The associations presented in Tables 2 and 3 did not change in the sensitivity analysis.

## Discussion

The effects of demographic, socioeconomic, self-reported health, and lifestyle factors were examined in this prospective cohort study on self-reported MSCs after 13 years of follow-up in the population of Tromsø, Northern Norway. The odds of having MSCs in any body region were 46% higher in women than men. The strong association between female gender and MSCs is broadly supported by other studies [2, 6, 22, 34], including our previously published cross-sectional study [25]. Despite this, the present study did not reveal effect differences in predictors that could explain the higher prevalence of MSCs in women. On the contrary, mental health complaints was an important risk factor for MSCs in men. These findings could indicate that other unmeasured factors are contributing substantially to the gender differences in the development of MSCs.

Low educational level, followed by poor self-perceived health were the strongest predictors in the regression analyses of overall MSCs (mild/severe MSCs versus no MSCs), and they were also important predictors in the analyses of individual body regions. This emphasises findings of previous studies examining how socioeconomic status and poor self-perceived health associates to MSCs [11, 14, 25, 35]. However, the size of the associations was smaller in the present study than in our previous cross-sectional study, which might imply that low educational level and poor self-perceived health are both predicting subsequent MSCs, but can also be consequences of established MSCs. Socioeconomic status and self-perceived health are predictors of several other diseases [36] and mortality [37, 38]. However, it is hard for prevention programs to directly intervene on these factors at an individual level; instead, results from the current study helps clinicians outline individuals at risk. Moreover, MSCs are a major and costly public health problem, affecting a large portion of the general population. Policymakers should pay particular attention to the highlighted associations with socioeconomic and general health factors when providing preventive health services in the community.

The results of our gender-stratified analyses of physical activity showed an association with overall MSCs, but only among women in age-adjusted models. Moreover, these models showed a dose-response relationship with a significant trend. However, after adjustment for other covariates, the significant association did not persist in multivariable analyses for any level of physical activity. Neither did region-specific analyses add convincing evidence of a relationship between physical activity level and MSCs. Thus, we were not able to unambiguously show that low physical activity level increases the risk of MSCs, which is in accordance with the cross-sectional study based on the Tromsø study (2007–08) [25], but in conflict with other prospective studies [9, 18, 39, 40]. In a cross-sectional study of a general Italian population, Cimmino et al could not reveal a correlation between physical activity and joint pain or swelling in a univariable analysis [41]. They did however, report that intense physical activity was related to joint pain in their multivariable analysis, while joint swelling was differently distributed in women and men exposed to intense physical activity. Thus, the relationship between physical activity and MSCs is likely to be complex. Examining associations between MSCs and type of mechanical strain through job or leisure time, not only level of leisure time physical activity, would possibly provide further knowledge, and could nuance these findings.

Mundal et al used the hospital anxiety and depression scale (HADS) to assess mental health complaints, and found that it predicted the onset of chronic widespread MSCs in a study with a similar design [16]. HADS and CONOR-MHI have been found to correlate well [30]. Although, we do not have MSCs data that meets the criteria of chronic widespread MSCs [42], our study, together with the Mundal et al study strongly suggests that patients with mental health complaints (i.e. symptoms of depression and/or anxiety) are prone to develop symptoms from the musculoskeletal system. Furthermore, our analyses are somewhat more detailed, and the current study indicates that mental health complaints may have stronger implications for some region-specific MSCs, especially lumbar back complaints, and mostly among men. This is an interesting finding for which the underlying mechanisms are not obvious and future studies should aim to further explore such associations.

It is broadly accepted that smoking is associated with MSCs [10, 12, 19, 43]. Unfortunately, baseline smoking information in our study did not include information on former smoking. However, our main goal in including the smoking variable was to explore if current smoking at baseline influenced men and women differently, as has been previously reported [25, 26]. We found a somewhat stronger association for current smoking and MSCs in women than in men, but the difference was not significant. The current study therefore suggests a temporal relationship between smoking and MSCs without gender differences. The significant association between baseline smoking and subsequent MSCs 13 years later could have been influenced by changes of smoking status during the follow-up time. Most smokers in Norway start smoking before the age of 20 years [44]. By including inhabitants older than 25 in this study, it is likely that those changing smoking status during the follow-up time actually stopped smoking. Therefore the significant association between smoking and MSCs found in the current study is more likely to be diluted than inflated. Future studies should aim to explore if stopping smoking reduces the risk of subsequent MSCs later in life.

Both overweight and obesity were significantly predicting MSCs in our study. The strongest association with obesity was found in the lower extremities, increasing the odds of MSCs by 74%. The suggested underlying mechanisms of this are multi-factorial [45–48]. A cross-sectional study from the Netherlands found a dose-response relationship between increasing BMI and MSCs in the lower extremities [49]. These findings fit the hypothesis of mechanical exertion on muscles and joints as an important etiological factor of chronic MSCs in the lower extremities. Nevertheless, our finding of a significant association between obesity and arm/hand MSCs indicates that mechanisms other than mechanical exertion might contribute to this association.

Kvalheim et al explored the relationship between female reproductive health and chronic widespread MSCs in their study on a general population [50]. They found that early menarche was associated with 26% higher odds of MSCs, indicating that hormonal factors could contribute to the development of MSCs, as in other pain conditions [51], and suggested that this factor could partly explain the female dominance. Female reproductive health variables were not included in the present study. However, the relationship between MSCs and hormonal factors is likely complex, and proper examination would warrant a separate study.

### Strengths and limitations

The major strengths of this study are the prospective design and the inclusion of MSCs-free subjects at baseline. Thus, we can be certain that the risk factors developed before, and not as a consequence of the outcome. The baseline characteristics included in this study were chosen based on previous findings from the Tromsø study [25]. Although the follow-up design of the present study permits the assumption of temporality, causal relationships cannot be determined since temporality is only one of several criteria for causality [52]. Furthermore, even though the current study was performed in an arctic part of northern Europe, many of the factors shown to predict MSCs were similarly reported in large-scale population-based studies from countries such as the Netherlands [22], UK [12, 48], Italy [41], Canada [43] and Australia [14]. This is a major strength as it supports the generalisability of the data of the Tromsø study, but maybe more important, it indicates that predictors of MSCs show similar patterns in different areas.

The prevalence of possible risk factors in our study sample changed as we chose to exclude a relatively large proportion of participants. This was due to incomplete follow-up data and difficulties in building an overall MSCs end-point variable. In sum, our study sample had lower levels of negative health determinants than did the excluded population. This may have biased our results as we included only those who fully understood the questionnaire and provided complete answers. It is likely that the complete-case approach to self-reported MSCs makes our results more conservative. However, the sensitivity analysis did not reveal any changes in the associations.

## Conclusion

In conclusion, the results of this study helps clinicians outline individuals at risk of developing musculoskeletal complaints as it has demonstrated that several factors are predicting the onset of both mild and severe MSCs. With the prospective design, large study population, and the assumption of temporality, the present study indicates that, although not large, there are some differences between men and women regarding the effects of risk factors on the development of MSCs. However, no specific risk factors that could explain the higher prevalence of MSCs in women were revealed. This warrants further research to focus on other factors that could influence gender differences in the development of MSCs.

## Compliance with ethical standards

### Ethical approval

All procedures performed in studies involving human participants were in accordance with the ethical standards of the institutional and/or national research committee and with the 1964 Helsinki declaration and its later amendments or comparable ethical standards.

## Supporting information

S1 Appendix Sensitivity analysis by age- and gender-adjusted logistic regression analysis including all 6,415 participants reporting no MSCs^1^ at baseline in a general population of Norway^2^. Those reported MSCs at any body region were included as having MSCs, and those who did not report MSCs were coded as no MSCs.
^1^Musculoskeletal complaints (Mild/severe pain and/or stiffness in muscles and joints lasting at least 3 months during the past year), ^2^The Tromsø Study; a prospective study of an arctic general population consisting of 4,496 adult men and women free of MSCs at baseline, ^3^Binary regression, adjusted for age and gender (bold text = significant result), ^4^Cohort of Norway Mental Health Index ≥2.15, OR: odds ratio,
*significant interaction with gender; p<0.05.
(DOCX)

S2 Appendix Sensitivity analysis by multivariable logistic regression analysis including all 6,415 participants reporting no MSCs^1^ at baseline in a general population of Norway^2^. Those reported MSCs at any body region were included as having MSCs, and those who did not report MSCs were coded as no MSCs.
^1^Musculoskeletal complaints (Mild/severe pain and/or stiffness in muscles and joints lasting at least 3 months during the past year), ^2^The Tromsø Study; a prospective study of an arctic general population consisting of 6,415 adult men and women free of MSCs at baseline, ^3^Multivariable logistic regression analyses, the models included all variables listed *(bold text = significant result)*, ^4^Cohort of Norway Mental Health Index ≥2.15, OR: odds ratio. Interaction term analyses did not reveal significant gender interactions in this multivariable logistic regression analysis, p<0.05.
(DOCX)
