# Supplementary material for: A prospective cohort study on risk factors of musculoskeletal complaints (pain and/or stiffness) in a general population. The Tromsø study
Source: PLoS One. 2017 Jul 20;12(7):e0181417. doi: 10.1371/journal.pone.0181417 (PMC5519093; doi:10.1371/journal.pone.0181417)
Supplement: S1 Appendix — Those reported MSCs at any body region were included as having MSCs, and those who did not report MSCs were coded as no MSCs. 1Musculoskeletal complaints (Mild/severe pain and/or stiffness in muscles and joints lasting at least 3 months during the past year), 2The Tromsø Study; a prospective study of an arctic general population consisting of 4,496 adult men and women free of MSCs at baseline, 3Binary regression, adjusted for age and gender (bold text = significant result), 4Cohort of Norway Mental Health Index ≥2.15, OR: odds ratio, *significant interaction with gender; p<0.05. (DOCX) [file pone.0181417.s001.docx]

|  | **Odds ratio^3^ (95% Confidence interval)** | | |
| --- | --- | --- | --- |
|  | **Total** | **Men** | **Women** |
| Age (5 years age groups) | **1.01 (1.00-1.01)** | **1.01 (1.00-1.02)** | **1.01 (1.00-1.01)** |
| Gender (Women vs men) | **1.40 (1.25-1.53)** |  |  |
| Marital status (not married vs married) | 1.00 (0.88-1.10) | 1.02 (0.87-1.19) | 0.95 (0.82-1.11) |
| Current smoking (yes versus no) | **1.30 (1.20-1.45)^*^** | **1.24 (1.07-1.44)** | **1.37 (1.17-1.61)** |
| Self-perceived general health (poor vs good) | **1.68 (1.43-1.98)^*^** | **1.75 (1.40-2.20)** | **1.60 (1.26-2.04)** |
| Mental health complaints ≥2.15^4^ | **1.44 (1.10-1.91)** | **1.92 (1.23-3.01)** | 1.18 (0.83-1.70) |
| **Educational level** |  |  |  |
| Primary/secondary | **1.82 (1.59-2.10)** | **1.75 (1.50-2.10)** | **1.93 (1.59-2.35)** |
| Technical school | **1.56 (1.40-1.80)** | **1.55 (1.31-1.84)** | **1.59 (1.31-1.93)** |
| High school | 1.12 (0.93-1.40) | 0.95 (0.72-1.25) | 1.28 (0.99-1.65) |
| College/university | 1.00 | 1.00 | 1.00 |
| **Body mass index (kg/m^2^)** |  |  |  |
| ≤24.9 | 1.00 | 1.00 | 1.00 |
| 25.0-29.9 | **1.30 (1.15-1.43)** | **1.16 (1.00-1.40)** | **1.46 (1.23-1.73)** |
| ≥30 | **1.50 (1.23-1.80)** | **1.44 (1.11-1.90)** | **1.53 (1.15-2.03)** |
| *p-value for trend* | **p < 0.001** | **p = 0.001** | **p < 0.001** |
| **Physical activity** |  |  |  |
| Sedentary | 1.20 (0.92-1.57) | 1.04 (0.73-1.47) | **1.55 (1.01-2.38)** |
| Low | 1.14 (0.94-1.38) | 0.99 (0.78-1.30) | **1.45 (1.06-1.99)** |
| Moderate | 1.03 (0.85-1.25) | 0.94 (0.74-1.20) | 1.30 (0.92-1.72) |
| High  *p-value for trend* | 1.00  **p < 0.001** | 1.00  **p < 0.001** | 1.00  **p < 0.001** |
