# Supplementary material for: A prospective cohort study on risk factors of musculoskeletal complaints (pain and/or stiffness) in a general population. The Tromsø study
Source: PLoS One. 2017 Jul 20;12(7):e0181417. doi: 10.1371/journal.pone.0181417 (PMC5519093; doi:10.1371/journal.pone.0181417)
Supplement: S2 Appendix — Those reported MSCs at any body region were included as having MSCs, and those who did not report MSCs were coded as no MSCs. 1Musculoskeletal complaints (Mild/severe pain and/or stiffness in muscles and joints lasting at least 3 months during the past year), 2The Tromsø Study; a prospective study of an arctic general population consisting of 6,415 adult men and women free of MSCs at baseline, 3Multivariable logistic regression analyses, the models included all variables listed (bold text = significant result), 4Cohort of Norway Mental Health Index ≥2.15, OR: odds ratio. Interaction term analyses did not reveal significant gender interactions in this multivariable logistic regression analysis, p<0.05. (DOCX) [file pone.0181417.s002.docx]

|  | **Odds ratio^3^ (95% Confidence interval)** | | |
| --- | --- | --- | --- |
|  | **Total** | **Men** | **Women** |
| Age (5 years age groups) | 1.00 (1.00-1.01) | 1.01 (1.00-1.01) | 1.01 (1.00-1.01) |
| Gender (Women vs men) | **1.43 (1.28-1.60)** |  |  |
| Current smoking (yes versus no) | **1.22 (1.09-1.40)** | **1.18 (1.01-1.40)** | **1.25 (1.05-1.48)** |
| Self-perceived general health (poor vs good) | **1.54 (1.29-1.83)** | **1.60 (1.30-2.02)** | **1.48 (1.14-1.92)** |
| Mental health complaints ≥2.15^4^ | 1.22 (0.91-1.64) | **1.70 (1.05-2.64)** | 1.00 (0.70-1.45) |
| **Educational level** |  |  |  |
| Primary/secondary | **1.65 (1.43-1.90)** | **1.60 (1.32-1.93)** | **1.71 (1.40-2.11)** |
| Technical school | **1.52 (1.33-1.73)** | **1.53 (1.28-1.82)** | **1.51 (1.23-1.84)** |
| High school | 1.10 (0.90-1.30) | 0.91 (0.70-1.20) | 1.20 (0.92-1.54) |
| College/university | 1.00 | 1.00 | 1.00 |
| **Body mass index (kg/m^2^)** |  |  |  |
| ≤24.9 | 1.00 | 1.00 | 1.00 |
| 25.0-29.9 | **1.26 (1.12-1.41)** | **1.20 (1.00-1.40)** | **1.41 (1.18-1.70)** |
| ≥30 | **1.39 (1.14-1.70)** | **1.39 (1.07-1.81)** | **1.40 (1.02-1.83)** |
| *p-value for trend* | **p < 0.001** | **p = 0.001** | **p < 0.001** |
| **Physical activity** |  |  |  |
| Sedentary | 0.92 (0.70-1.22) | 0.82 (0.60-1.20) | 1.16 (0.74-1.83) |
| Low | 1.10 (0.90-1.30) | 0.94 (0.73-1.20) | 1.30 (0.92-1.80) |
| Moderate | 1.04 (0.85-1.30) | 0.94 (0.74-1.20) | 1.22 (0.88-1.70) |
| High | 1.00 | 1.00 | 1.00 |
| *p-value for trend* | p = 0.937 | p = 0.482 | p = 0.332 |
